# Applying the Lessons of Physiological Cell Culture to Human Embryo Culture for In Vitro Fertilization

**DOI:** 10.3390/biom16050618

**Published:** 2026-04-22

**Authors:** Abigail Pokorski, Ricardo Alva, Jacob E. Wiebe, Jeffrey A. Stuart

**Affiliations:** Department of Biological Sciences, Brock University, St. Catharines, ON L2S 3A1, Canadaralvaoropeza@brocku.ca (R.A.); jwiebe@brocku.ca (J.E.W.)

**Keywords:** physiological cell culture, amino acids, glucose, growth media, development, oxygen, hyperoxia

## Abstract

Growth media for human cell culture were developed in the twentieth century, when the first immortal human cell lines were established. The nutrient compositions of these media arose not from a desire to reproduce the microenvironment of the cells in vivo, but rather to encourage continuous replicative growth. Armed with comprehensive datasets detailing the metabolomes of the various fluid compartments within which cells reside, cell culturists are now exploring the effects of media designed to reproduce the in vivo environment on cell biology. The early results of this research indicate the media composition has profound impacts on cell form and function. In parallel, taking care to maintain oxygen at the relatively low levels found in vivo also affects many cellular activities. The lessons learned from ‘physiological cell culture’ should be applied to the culture of human embryos in the in vitro fertilization (IVF) clinic, where a critical stage of growth and development might be best supported by recreating, to the greatest extent possible, the environment of the oviduct and uterus. In this review, we translate recent advancements in physiological cell culture to emerging approaches in human embryo culture.

## 1. Introduction

Over the past 20 years, there has been a significant increase in the use of assisted reproductive technologies (ARTs), particularly in vitro fertilization (IVF), amongst couples experiencing infertility. In 2018, Canada saw over 16,000 IVF cycles performed, resulting in nearly 2500 pregnancies (Canadian Fertility and Andrology Society, 2019) [1]. Optimizing the success rates of this technique is an important objective of current reproductive medicine research.

Normally, an oocyte is released from an ovary and fertilized in the oviduct, where it develops into a cleavage-stage embryo, enters the uterus, and continues development into a blastocyst. The blastocyst implants in the uterus where it develops into a fetus. In IVF, oocytes are retrieved directly from the ovaries and then fertilized in a petri dish where they are maintained in a temperature-, humidity-, and gas-controlled incubator for typically 5–6 days prior to uterine implantation. Prior to uterine transfer and hopeful implantation, the fertilized egg grows, divides, and develops into a blastocyst with 70–100 cells. The quality of the blastocyst is of utmost importance to the success of the pregnancy—higher-quality blastocysts have a greater chance of becoming viable pregnancies [2]. For example, in a recent large study of 10,000 women undergoing a single blastocyst transfer, low-quality blastocysts resulted in a live birth 30% of the time, compared to 44% for higher-quality blastocysts [3]. Thus, blastocyst quality has immediate effects on the likelihood of a successful pregnancy; on the other hand, the longer-term consequences of lower-quality blastocysts that develop into a fetus are not well understood.

The early stages of embryo development are thus an important and sensitive time, during which it is essential to provide optimal conditions for development. It is during this stage of development that the maternal-to-zygotic transition (MZT) occurs, as maternal mRNA transcripts are gradually degraded and replaced by zygotic ones [4]. IVF culture conditions can influence the MZT by, for example, affecting the orchestration of DNA methylation events and thus gene expression [5]. Culture conditions also impinge upon nutrient sensing and signaling pathways that influence embryonic development. There is evidence that human embryos, as has been documented in many other mammalian species, undergo a growth-arrested diapause mediated by mTOR inhibition when their microenvironment is not conducive to development [6]. mTOR, the mechanistic target of rapamycin, is a key growth signaling node that is regulated by energy and amino acid availability and promotes a range of anabolic activities including protein synthesis [7]. Uterine implantation is compromised under such conditions, an obviously undesirable outcome for IVF. Thus, the details of embryo culture are critical to its future development.

## 2. The Advent of Physiological Cell Culture

Many mammalian cell biologists who routinely use cell culture as an experimental approach have begun to transition toward using more physiologically representative media and oxygen levels, particularly following the recent publication of several studies describing effects of non-physiological media (e.g., DMEM) on most aspects of cell function [8,9,10]; reviewed in [11]. Although most of this work has been done in cancer cell lines, where applied in relevant reproductive contexts, the impact is similar [12]. In general, the implications of physiological cell culture likely apply to any cell type, including those of the developing embryo. The purpose of this literature review is to examine the practice of embryo culture in IVF in the light of our increasing knowledge of how the nutrient composition of growth media modulates gene expression and instigates the ‘metabolic re-wiring’ of cells.

The goal of physiological cell culture is to mimic the characteristics of the in vivo microenvironment, an elusive goal since this is incredibly complex and dynamic. Two aspects that are generally more readily addressed, however, are growth medium composition and oxygen levels. We will first consider medium composition.

Most of the growth media used in routine culture of cells, including cancer cells, myoblasts, and fibroblasts, were developed in the mid-20th century. These media, including Dulbecco’s Modified Eagles Medium (DMEM) and Roswell Park Institute (RPMI) 1640, continue to be used today. However, in 2017, Human Plasma-Like Medium (HPLM) was developed as a physiological medium modeled on the human blood plasma metabolome. Cancer cell lines growing in HPLM were shown to have broadly altered transcriptomes and re-wired metabolism that affected their susceptibility to the chemotherapy drug 5-fluorouracil [13]. In 2019, Plasmax was developed as a more complete (i.e., more individual metabolites) plasma-like medium. Growth of several cancer cell lines in Plasmax versus standard cell culture media had broad effects, including reducing the pseudohypoxia artifact associated with non-physiological pyruvate concentrations in DMEM-F12. We have shown effects of Plasmax (versus DMEM) on cellular bioenergetics and mitochondrial network dynamics [9]. We recently developed a Tumour MicroEnvironment Medium (TMEM) with a composition based on the interstitial fluid metabolome of murine pancreatic ductal adenocarcinoma (PDAC). Growth of PDAC cells in TMEM and typical tumour hypoxia (1.5% O_2_) versus a plasma-like medium and physiologically normal oxygen levels (5% O_2_) again showed broad effects, encompassing the transcriptome, growth, and energy metabolism [14]. Growth of PC-3 prostate cancer cells in TMEM versus DMEM significantly altered their susceptibility to the chemotoxin docetaxel (unpublished observations). In summary, media composition consistently affects a wide range of cellular activities. Culturing cells in physiologically relevant nutrient conditions will improve the reproducibility and translatability of research, and its benefits may extend to embryo culture.

## 3. The In Vivo Environment of the Embryo

Any effort to reproduce an in vivo microenvironment in vitro requires that consideration be given to the nutrient metabolome. In this case, the relevant in vivo microenvironments to model in vitro are oviduct fluid and uterine fluid. There is surprisingly little data on the detailed metabolite composition of oviductal fluid. The most complete dataset for human oviduct fluid comes from the recent study by Utsunomiya et al. [15], who measured inorganic salts, glucose, organic acids, and amino acids in 28 females between 26 and 40 years of age (Table 1).

## 4. Issues with Current Commercially Provided Embryo Culture Media

The paucity of detailed metabolomic data for oviduct fluid has been a limitation in IVF. Perhaps equally important, however, is the reluctance of commercial providers of embryo culture media to disclose detailed formulations of their products [16,17]. IVF clinics generally use commercial media, and the companies that make and provide these have not shared their detailed formulations. This has happened despite evidence from some clinical trials that some of these commercial media may perform better than others (reviewed in [18]). As a result, it has previously been virtually impossible to assess the physiological relevance of existing media in terms of their similarity to oviduct fluid. This alarming information gap has been the topic of recent editorials [16].

Ultimately, the situation has been addressed not by voluntary disclosures of formulations but rather by extensive independent metabolomic analyses of human embryo culture media by Tarahomi et al. [19] and Zagers [17]. In the latter, 40 components of commercial embryo culture media, with and without recommended supplements, were identified and quantified. A total of 47 commercially available media marketed as being appropriate for fertilization, cleavage-stage, or blastocyst-stage human embryo culture were included in this study. Several of these were ‘continuous media’, marketed for use throughout the duration of embryo culture. In order to appropriately interpret the formulation of the various human embryo culture media, the human embryo developmental stages were aligned with menstrual cycle phases and anatomical locations previously established in the literature. Fertilization occurs in the ampulla of the oviduct, around midcycle, just following ovulation [20]. Therefore, we compared the compositions of select commercial fertilization media to the composition of human oviduct fluid during midcycle, around day 14 [15]. Similarly, since early cleavage occurs in the isthmus of the oviducts during the early luteal phase [20], the compositions of cleavage media were compared to human oviduct fluid at the luteal phase, around days 15–17 [15]. The blastocyst then reaches the uterus and implants on the uterine wall during the mid-to-late luteal phase [20]. Therefore, we compared human embryo blastocyst media to the composition of human uterine fluid [21]. These comparisons are shown in Table 2, Table 3 and Table 4 below, which are examples taken from many such analyses in [17].

Two trends are evident in the data. Firstly, the various commercial media marketed for use in any specific developmental stage vary widely in their compositions. While blastocyst-stage media tend to be more uniform amongst the examples shown here, the fertilization-stage media have little in common with each other (Table 2). Similar variability is seen amongst cleavage-stage media (Table 3). Secondly, the compositions of fertilization or cleavage-stage media do not closely resemble that of oviductal fluid, while blastocyst-stage media are generally reasonable approximations of uterine fluid.

## 5. Culture Media Metabolites Affect Intracellular Signaling and Gene Expression

There are numerous issues with the use of non-physiological growth media in human embryo culture. Some researchers and clinicians have advocated for the ‘let the embryo decide’ approach, i.e., providing nutrients in excess and allowing the developing embryo to take up those it needs [22]. However, recent research on physiological cell culture has shown that the result of this approach is a complex cellular-level adaptive response manifesting as changes to the transcriptome, rates of cell division, epigenetic regulation, and energy metabolism (reviewed in [11,23,24]). Indeed, virtually all cell properties that have been measured are affected by the composition of growth media. Below we discuss several examples of specific nutrient concentrations affecting cell biology that may impinge on the growth and development of the embryo.

Surprisingly, high levels of the D-lactate enantiomer were found in some commercial embryo culture media (Table 3 and Table 4). For example, it comprised ~50% of the total lactate found in Early Cleavage media and MultiBlast Blastocyst media from Irvine Scientific. D-lactate, the stereoisomer of L-lactate, cannot be metabolized to produce ATP to support embryonic development. In fact, a study by [19] found a correlation between elevated D-lactate levels and reduced cleavage rate, impaired blastocyst formation, and compromised implantation potential. Importantly, while histone lysine L-lactylation is thought to play a role in embryonic development [25,26], it is currently unclear whether D-lactate contributes to histone lactylation in this context. Irvine Scientific recently discontinued their Early Cleavage and MultiBlast media.

In the first several days post-fertilization, L-lactate appears to be an important energy source for the developing embryo (reviewed in [27]), whereas by the blastocyst stage it is a key metabolic end-product with intercellular signaling roles [28]. The lactate concentration in human oviduct is 5–10 mM (Table 1; [29]), which is appreciably higher than in blood plasma (1–2 mM; [30]). While the fertilization-stage media shown in Table 2 have similar L-lactate concentrations, cleavage-stage media range from 2.2 to 47.5 mM. Higher concentrations of L-lactate during the cleavage stage may have metabolic effects, including on the metabolism of pyruvate [31]. Moreover, L-lactate has roles in signaling and epigenetic regulation that may be of particular importance in the early embryo. Extracellular L-lactate concentration can affect epigenetic reprogramming during embryo development via histone lactylation [32,33,34]. Lactylation of histone lysine residues is an important epigenetic mechanism that links cellular metabolism to development. Aberrant lactylation has the potential to dysregulate key developmental processes, such as zygotic genome activation [35].

The suite of amino acids, and their individual concentrations, found in embryo culture media is particularly displaced from those found in oviduct and, to a lesser extent, uterine fluids. Essential amino acids play critical roles in protein synthesis, hormone production, neurotransmitter synthesis, immune function, energy regulation, and nutrient sensing. For example, leucine plays a key role in nutrient sensing via mTOR, impinging on the protein synthesis needed for cell division and differentiation [7]. Iyer et al. [6] recently demonstrated the role of mTOR in human blastocyst-stage development. Leucine is essentially absent in many fertilization- and cleavage-stage media, though it is much closer to physiologically relevant levels in blastocyst media. In general, the amino acid profile of early-stage culture media has particularly non-physiological amino acid compositions, in many cases.

## 6. Towards More Physiologically Representative Embryo Culture Media

The issue of physiological human embryo culture media has recently been addressed by Utsunomiya et al. [15], who developed OVIT medium based on the oviduct fluid metabolomic data listed above. Although the entire formulation was not provided by the authors, the amino acid concentrations are similar to the measured mean values of oviduct fluid (pooled) from Table 1 above. In a clinical trial including several thousand people, growth of embryos in this medium resulted in higher quality blastocysts for transfer than several commercial media. This outcome was particularly pronounced in patients with a maternal age of 38 and greater. Thus, this new physiological medium may be the best available option for human embryo culture yet.

One important consideration in embryo culture, as in any cell culture using physiological media, is the rate of nutrient depletion/waste accumulation. This is particularly relevant in continuous culture media, where the composition on day 5 may differ markedly from that on day 0 due to the continuous uptake of nutrients and excretion of metabolic wastes. We have recently reported rates of nutrient depletion from Plasmax by human cancer cells, showing that significant nutrient depletion can occur within 24 h in culture, necessitating daily media exchanges [36]. Rates of amino acid utilization from media have been evaluated in human embryo culture at blastocyst stage [37]. Leucine is consumed at a rate of about 5 pmol/embryo/h, or about 240 pmol/embryo over two days. For culture in 25 µL of medium with 185 µM leucine (total 4625 pmol leucine), this is only about 5% depletion and therefore unlikely to perturb signaling systems. However, for cleavage-stage culture with extremely low (or zero) concentrations of individual amino acids, depletion is possible and should be similarly assessed.

Another important consideration for the implementation of physiological embryo culture is the choice of sequential or continuous culture regimes, which differ with respect to how closely they attempt to recapitulate the changing nutrient environment of the developing embryo. The sequential approach involves the use of two chemically distinct media: one cleavage-stage medium, used for the first three days of culture, and one blastocyst-stage medium, used from day 3 onward. These media contain distinct metabolite profiles and seek to emulate the nutrient environment of the embryo across both stages. Cleavage-stage media, for example, are generally lower in their supply of amino acids and glucose, while including high levels of pyruvate and lactate, preferred metabolic fuels in early stages of development [38] (see Table 3). Blastocyst-stage media, on the other hand, are higher in their supply of amino acids and glucose (see Table 4). The adoption of separate culture media for each developmental stage ensures that culture conditions are tailored to suit the relatively quiescent metabolic state of the cleavage-stage embryo and the more highly glycolytic, biosynthetic state of the embryo in the blastocyst stage.

The continuous approach, on the other hand, involves the culture of embryos in single culture media that contain the entire range of nutrients needed for the duration of in vitro development. An example of this would be the OVIT culture medium described by Utsunomiya et al. [15], which uses pooled metabolomic data from human oviductal fluid in the midcycle and luteal phases to approximate the nutrient environment of the developing embryo. While this approach has some limitations, including the potential for nutrient depletion and waste accumulation, as well as the need to include nutrients at concentrations that may be inappropriate for individual developmental stages, its advantages include the ability to reduce handling and environmental disturbance, thereby maintaining more stable culture conditions and minimizing stress associated with temperature shifts, pH fluctuations, and repeated removal from the incubator.

Multiple laboratories have sought to investigate the outcomes associated with sequential versus continuous culture regimes, with respect to both blastocyst formation, embryo quality, and pregnancy/miscarriage rates; however, results have been conflicting and seem to depend on the outcome being measured. For example, while studies from Reed et al. [39] and López-Pelayo [40] observed that continuous culture media produce more high-quality embryos and increase blastocyst formation in vitro, a meta-analysis from Dieamant et al. [41] found that the choice of sequential or continuous media makes no difference to pregnancy and miscarriage rates. More recently, a study from Stimpfel et al. [42] found that continuous media produce fewer poor-quality embryos on day 3 of culture, but found no statistically significant effects with respect to pregnancy or live birth rates. Therefore, while there is certainly evidence to suggest that the choice of culture regime has significant effects on embryo quality during in vitro development, it is less clear whether these differences translate into meaningful improvements to clinical outcomes such as pregnancy or live birth rates. Future research in this field will certainly benefit from the adoption of standardized measures of embryo quality that evaluate the embryo’s epigenetic and metabolic states rather than relying solely on morphological or downstream clinical endpoints.

## 7. Oxygen Is a Key Variable in Embryo Culture

Most mammalian tissue cells exist in oxygen levels of 2–9% in vivo [43]–much lower than atmospheric levels of ~20.5%. Most standard CO_2_ incubators for mammalian cell culture do not regulate O_2_ levels, which equilibrate to ~18.5% due to the addition of 5% CO_2_ and water vapour. Thus, most mammalian cell culture for research purposes is performed under hyperoxic conditions. This has wide-ranging deleterious effects on cells, including increased reactive oxygen species production, increased oxidative DNA damage, reduced cell replication rates, and early onset replicative senescence [23]. It is therefore important to consider the exposure of human embryos to oxygen during in vitro culture.

Oxygen levels experienced by the embryo in vivo range from 5–7% in oviduct fluid to ~2% in the uterine cavity [44,45]. The effects of oxygen tension during mammalian embryo culture have been well studied over several decades. Numerous studies have reported that culture under low oxygen tension (typically 5–6% O_2_) improves embryo development and quality, including embryo morphology, blastocyst formation, and embryo utilization, and some studies have also reported improved implantation, pregnancy, cumulative pregnancy, or live birth outcomes relative to atmospheric oxygen [46,47,48,49,50,51,52,53]. However, these clinical benefits have not been observed consistently across studies. Some randomized studies reported improved embryo-quality-related endpoints without corresponding gains in ongoing pregnancy or live birth, and meta-analyses suggest that the evidence for improved clinical outcomes remains insufficient or of low certainty [50,51,54,55,56]. It is important to note that any effect of oxygen tension on clinical outcomes may be obscured by factors downstream of culture itself, since implantation and pregnancy depend not only on embryo competence but also on endometrial receptivity, embryo-transfer technique, and patient-specific factors [57]. In addition, oxygen is only one component of the culture system, and its effects may interact with other laboratory variables, including medium formulation and protein supplementation. Importantly, the absence of a clear difference in pregnancy or perinatal endpoints should not be interpreted as evidence that atmospheric and low-oxygen culture exert equivalent effects on the embryo, because atmospheric oxygen constitutes a hyperoxic condition relative to the in vivo environment, and its longer-term developmental consequences remain insufficiently defined. Consistent with this rationale, the European Society of Human Reproduction and Embryology (ESHRE) guidelines recommend culture in low oxygen tension in their current guidelines [58].

A further limitation of low-oxygen culture is that oxygen tension is usually defined by the incubator headspace rather than measured in the medium immediately surrounding the embryo. Thus, the oxygen concentration actually experienced by the embryo may differ from the nominal incubator setting and is likely shaped by factors such as medium volume, diffusion distance, oil overlay, embryo density, and developmental stage. As embryonic oxygen consumption increases during development, the local microenvironment may diverge further from the nominal gas-phase oxygen tension over time. Evidence from mouse and bovine embryos cultured in ambient air shows that oxygen gradients can form in the medium immediately surrounding the embryo and become more pronounced with developmental stage [59,60]. Thus, direct assessment of oxygen at the embryo level will be crucial for optimizing physiologically relevant culture conditions.

The type of incubator may have implications for physiological embryo culture. Where the developing embryo must be removed from the incubator repeatedly for morphological assessment under a microscope, fluctuations in temperature, CO_2_, pH, and O_2_ levels are expected to occur. Incubators that can image the embryos in situ would be expected to have greater stability. Kermack et al. [37] studied this with 585 sibling zygotes. 289 were cultured in incubators with self-contained imaging (time-lapse system; TLS), such that embryos were not removed for assessment. A total of 296 zygotes were incubated in standard incubators requiring them to be periodically removed for assessment. Interestingly, more zygotes cultured in TLS developed to the blastocyst stage (55%) versus 45% in standard incubators. Similarly, there were more high-quality embryos from culture in TLS incubators (31% versus 23%). Other studies of this nature have produced a variety of confirmatory or contradictory results [61,62,63,64,65,66]. Differences between studies may relate to time spent out of the incubator or other aspects of a clinic’s workflow. Further research must be done to confirm whether a certain type of incubator yields better blastocyst quality, but it is likely useful to avoid any environmental stress where possible.

## 8. Additional Considerations and Future Directions

### 8.1. Proteinaceous Sources in Embryo Culture Media

An additional and often underappreciated source of complexity in embryo culture media is protein supplementation. In routine IVF practice, media are commonly used with proteinaceous supplements such as human serum albumin (HSA) or more complex serum-derived supplements [67,68]. These additives can support embryo culture by binding or carrying lipids, metabolites, growth factors, and other bioactive molecules [67], but they are also among the least defined components of the culture system. Analyses of protein supplements used for human embryo culture have shown substantial compositional variability, including previously undescribed components and differences in metal content. Morbeck et al. noted high concentrations of pro-oxidant transition metals in many of these supplements, which may interact with oxygen and affect blastocyst development [68]. Broader analyses of commercial embryo media confirm that supplementation contributes further to the heterogeneity of the final formulations used in practice [17,68].

From the standpoint of physiological embryo culture, this is important because improving the ionic and metabolite composition of the base medium alone may not fully define the embryo microenvironment if the accompanying protein supplement remains variable or incompletely characterized. This concern may be especially relevant for albumin-based supplements, which can introduce batch-dependent molecular cargo into the culture system. Indeed, commercially available albumin preparations differ in their lipid composition, and these differences were associated with changes in embryo development, implantation, and fetal growth in a mouse model [69,70]. More standardized alternatives such as recombinant human albumin have supported embryo development comparably to plasma-derived HSA in prospective studies [68,71], although their bound molecular cargo may still differ depending on product formulation and should not be assumed to be physiologically representative.

### 8.2. Toward Dynamic Embryo Culture Systems

Static embryo culture in a microdrop is an imperfect representation of the in vivo environment. In vivo, the preimplantation embryo develops within a changing physical and chemical milieu as it moves through the oviduct and into the uterus. Accordingly, an important future direction is the development of dynamic culture systems, particularly microfluidic platforms, that permit tighter control of the embryo microenvironment over time and may allow a more physiologically relevant fluid movement, with nutrient renewal and waste removal, than conventional static culture [72,73]. Although such approaches remain under evaluation, they underscore that physiological embryo culture may ultimately require attention not only to medium composition and oxygen tension, but also to the physical culture platform itself.

### 8.3. Clinical, Translational, and Regulatory Barriers to Implementation

The implementation of more physiological embryo culture media in routine IVF practice will require more than evidence of altered embryo metabolism or improved preimplantation development. As we have discussed above, a major translational barrier is that embryo culture media are often commercial proprietary products with incompletely disclosed formulations, which limits independent mechanistic evaluation, cross-study comparison, and rational optimization [16]. In parallel, any new medium must be introduced within the strict quality-management framework of the IVF laboratory, where media should be embryo-culture grade, appropriately regulated for clinical use, accompanied by batch-specific quality-control documentation, and locally validated when adequate testing is not provided by the manufacturer [58]. Together, these issues suggest that physiological media will require not only a strong biological rationale, but also transparency, manufacturing consistency, laboratory validation, and convincing clinical evidence before widespread adoption can be justified.

## 9. Conclusions

The recent development and commercialization of plasma-based media such as Plasmax and HPLM has encouraged more widespread efforts to improve the physiological relevance and translatability of cell culture models. Here, we have argued that human embryo culture for IVF is a field in need of similar considerations. Remarkably, IVF clinics generally use commercially developed media whose composition is not disclosed. The compositions of various commercial media for IVF vary greatly in their nutrient levels, and the vast majority do not resemble oviductal fluid at specific stages of embryonic development. The embryo is highly sensitive to environmental conditions, and the inclusion of non-physiological metabolites, such as D-lactate, or metabolites at non-physiological concentrations, will challenge and potentially disrupt metabolic and epigenetic processes occurring during development. The findings of Utsunomiya et al. clearly demonstrate the importance of this, as they showed that incorporating oviduct-like nutrient levels to culture media improved outcomes for IVF patients. Further, while it has already been established that culture in low oxygen tensions increases the production of high-quality embryos, efforts should be made to ascertain how oxygen levels in the incubator headspace are reflected in the medium at the embryo level. IVF is perhaps the most important instance of physiological cell culture that should receive immediate attention in the light of recent findings from the broader cell biology community.

## Figures and Tables

**Table 1:** Amino acid composition of human oviduct fluid. Mean concentrations of constituents of human oviduct fluid collected between day 0 and day 12 from 28 females between the ages of 26 and 40. Modified from [15].

	Metabolite	Mid-Cycle Mean ± SEM (µM) (*n* = 21)	Luteal Phase Mean ± SEM (µM) (*n* = 7)	Pooled Mean ± SEM (µM) (*n* = 28)
**Inorganic Salts**	Sodium	156,137 ± 5587	153,223 ± 4411	155,352 ± 4291
	Potassium	15,832 ± 1609	13,723 ± 1056	15,264 ± 1223
	Calcium	1331 ± 120	1133 ± 86	1278 ± 92
	Magnesium	510 ± 59	599 ± 54	534 ± 46
	Chlorine	135,966 ± 5329	137,405 ± 5660	136,339 ± 4172
	Phosphate	2788 ± 467	1677 ± 198	2500 ± 361
**Carbohydrates and**	Glucose	3429 ± 351	6159 ± 635	4164 ± 381
**Organic Acids**	Pyruvate	209 ± 35	106 ± 23	182 ± 28
	Lactate	5193 ± 666	3136 ± 208	4660 ± 525
	Citrate	134 ± 10	178 ± 19	146 ± 9
**Essential Amino Acids**	Valine	115 ± 14	149 ± 19	124 ± 12
	Leucine	92 ± 14	95 ± 11	93 ± 10
	Isoleucine	40 ± 6	42 ± 6	41 ± 5
	Lysine	215 ± 31	160 ± 22	201 ± 24
	Threonine	126 ± 15	123 ± 19	125 ± 12
	Methionine	27 ± 4	21 ± 2	25 ± 3
	Histidine	58 ± 8	67 ± 12	61 ± 6
	Phenylalanine	50 ± 7	55 ± 6	51 ± 5
	Tryptophan	18 ± 2	29 ± 3	21 ± 2
	Arginine	133 ± 21	93 ± 14	123 ± 16
	Cystine	58 ± 13	45 ± 13	55 ± 10
	Tyrosine	54 ± 8	58 ± 8	55 ± 6
	Glutamine	426 ± 38	542 ± 44	455 ± 32
**Non-essential Amino**	Glycine	1114 ± 140	1137 ± 216	1120 ± 116
**Acids**	Alanine	335 ± 45	351 ± 47	339 ± 36
	Glutamate	670 ± 99	508 ± 69	630 ± 77
	Aspartate	142 ± 20	124 ± 19	137 ± 16
	Asparagine	16 ± 1	23 ± 6	18 ± 2
	Proline	123 ± 17	111 ± 16	120 ± 13
	Serine	196 ± 27	216 ± 55	201 ± 24
**Other**	Taurine	1732 ± 210	1265 ± 198	1615 ± 168

**Table 2:** Metabolite composition (µM) of human midcycle oviduct fluid vs. commercial fertilization and continuous culture media.

Supplier	Composition of Human Oviduct Fluid during Midcycle [15]	CookMedical Sydney IVF Fertilization Medium [17]	Vitrolife G-IVF Fertilization Medium + HSA [17]	CooperSurgical Quinn’s Advantage Fertilization Medium + HSA [17]	Vitrolife G-TL Continuous Culture Medium [17]
Glucose	3429 ± 351	3000	2510	2720	1020
L-lactate	5193 ± 666	4200	10,100	4300	9700
Pyruvate	209 ± 35	190	320	300	470
Histidine	58 ± 8	0	0	1	71
Isoleucine	40 ± 6	0	0	0	202
Leucine	92 ± 14	0	1	0	183
Lysine	215 ± 31	4	18	0	213
Methionine	27 ± 4	0	0	0	54
Phenylalanine	50 ± 7	0	1	10	97
Threonine	126 ± 15	0	0	0	41
Tryptophan	18 ± 2	3	4	1	26
Valine	115 ± 14	2	3	0	186
Arginine	133 ± 21	0	0	14	315
Glutamine	426 ± 38	114	0	0	22
Glycine	1114 ± 140	3740	38	125	208
Proline	123 ± 17	110	17	100	110
Tyrosine	54 ± 8	3	3	0	100
Alanine	335 ± 45	186	11	5	71
Aspartate	142 ± 20	113	105	112	146
Asparagine	16 ± 1	65	7	112	22
Glutamate	670 ± 99	105	117	4	130
Serine	196 ± 27	97	13	98	123

HSA = human serum albumin supplement.

**Table 3:** Metabolite composition (µM) of human luteal phase oviduct fluid vs. several commercial cleavage and continuous culture media.

Supplier	Human Oviduct Fluid During Luteal Phase [15]	CookMedical Sydney IVF Cleavage Medium [17]	Irvine Scientific Early Cleavage Medium + DSS [17]	InVitroCare IVC-TWO Cleavage Medium + HSA [17]	Vitrolife G-TL Continuous Culture Medium [17]
Glucose	6159 ± 635	310	450	380	1020
L-lactate	3136 ± 208	2200	8878	47,500	9700
D-lactate	N/A	0	10,422	0	0
Pyruvate	106 ± 23	190	280	60	470
Histidine	67 ± 12	7	0	0	71
Isoleucine	42 ± 6	18	191	0	202
Leucine	95 ± 11	21	0	0	183
Lysine	160 ± 22	25	0	0	213
Methionine	21 ± 2	4	0	0	54
Phenylalanine	55 ± 6	10	0	0	97
Threonine	123 ± 19	0	0	0	41
Tryptophan	29 ± 3	5	1	1	26
Valine	149 ± 19	20	0	0	186
Arginine	93 ± 14	26	0	0	315
Glutamine	542 ± 44	93	0	0	22
Glycine	1137 ± 216	3833	6	96	208
Proline	111 ± 16	106	0	125	110
Tyrosine	58 ± 8	18	2	2	100
Alanine	351 ± 47	157	0	78	71
Aspartic Acid	124 ± 19	108	4	82	146
Asparagine	23 ± 6	55	0	111	22
Glutamate	508 ± 69	104	0	97	130

DSS = Dextran Serum Supplement; HSA = human serum albumin supplement.

**Table 4:** Metabolite composition (µM) of human uterine fluid vs. several commercial blastocyst and continuous culture media.

Supplier	Composition of Human Uterine Fluid Mean ± SEM (µM) (n = 22) [21]	Vitrolife G-2 PLUS Blastocyst Medium [17]	CooperSurgical Quinn’s Advantage Blastocyst Medium + SPS [17]	Irvine Scientific Multi-Blast Medium + SSS [17]	Vitrolife G-TL Continuous Medium [17]
Glucose	5100 ± 1780	3260	2500	2710	1020
L-lactate	6600 ± 1120	5800	4000	8556	9700
D-lactate	N/A	0	0	10,040	0
Pyruvate	80 ± 20	80	90	140	470
Histidine	97 ± 17	78	67	65	71
Isoleucine	114 ± 14	198	178	164	202
Leucine	193 ± 31	185	169	181	183
Lysine	298 ± 53	204	181	202	213
Methionine	38 ± 9	57	47	45	54
Phenylalanine	1008 ± 15	100	102	111	97
Threonine	117 ± 20	43	136	140	41
Tryptophan	37 ± 4	26	28	30	26
Valine	190 ± 26	194	187	185	186
Arginine	136 ± 40	314	298	310	315
Glutamine	227 ± 40	109	0	9	22
Glycine	955 ± 156	110	126	112	208
Proline	221 ± 33	115	103	63	110
Tyrosine	86 ± 15	99	92	94	100
Alanine	513 ± 82	180	4	67	71
Aspartic Acid	230 ± 29	124	112	60	146
Asparagine	33 ± 7	47	81	35	22
Glutamate	1162 ± 183	114	6	63	130
Serine	220 ± 46	102	92	64	123

SPS = Serum Protein Substitute; SSS = Serum Substitute Supplement.

## Data Availability

No new data were created or analyzed in this study. Data sharing is not applicable to this article.
